# Trunk velocity-dependent Light Touch reduces postural sway during standing

**DOI:** 10.1371/journal.pone.0224943

**Published:** 2019-11-07

**Authors:** Anirudh Saini, Devin Burns, Darian Emmett, Yun Seong Song

**Affiliations:** 1 Department of Mechanical and Aerospace Engineering, Missouri University of Science and Technology, Rolla, Missouri, United States of America; 2 Department of Psychological Science, Missouri University of Science and Technology, Rolla, Missouri, United States of America; University of Valencia, SPAIN

## Abstract

Light Touch (LT) has been shown to reduce postural sway in a wide range of populations. While LT is believed to provide additional sensory information for balance modulation, the nature of this information and its specific effect on balance are yet unclear. In order to better understand LT and to potentially harness its advantages for a practical balance aid, we investigated the effect of LT as provided by a haptic robot. Postural sway during standing balance was reduced when the LT force (~ 1 N) applied to the high back area was dependent on the trunk velocity. Additional information on trunk position, provided through orthogonal vibrations, further reduced the sway position-metric of balance but did not further improve the velocity-metric of balance. Our results suggest that limited and noisy information on trunk velocity encoded in LT is sufficient to influence standing balance.

## Introduction

In Light Touch (LT), small interaction forces on a fingertip have been shown to improve human postural stability during standing [1–3]. Touching a stationary object with a fingertip using forces under 1 N was effective in reducing the center-of-pressure (CoP) sway in a wide range of populations including healthy young adults [4], older adults [5], patients with a stroke [6, 7], peripheral neuropathy[8], Parkinson’s Disease [9], or an anterior cruciate ligament (ACL) injury [10].

Due to the simplicity in providing LT and its effectiveness in improving standing stability, LT is believed to have the potential to become a useful balance aid [2]. The generally accepted idea is that LT does not provide direct mechanical support to increase dynamic stability due to the small forces involved [2, 11, 12], but instead it provides additional sensory information that participants can use to better guide postural adjustments. Indeed, LT may compensate for the loss of sensory inputs related to balance originating from the ACL [10], bottom of the foot [8], or vestibular system [12]. Nonetheless, it is still unclear what relevant balance information is encoded in the small interaction forces from LT [13–16]. At the very least, the improvement in stability due to LT strongly suggests that LT provides some form of balance information that the human brain understands and can act upon. In this view, LT may be regarded as a form of tactile biofeedback, the specific information it provides being yet unknown.

Unfortunately, traditional and widely used LT experimental methodologies may be inadequate to investigate how LT leads to improved stability, often in terms of reduced postural sway [1–10]. In these experiments, the measured LT force at the fingertip is affected by both the participants’ voluntary movement (e.g. arm and hand motion) as well as postural sway. A change in LT force could be due to postural sway and denote a useful signal for guiding balance, but it could also result from active modulation of the force itself by the participant. While the participant can have awareness of when changes are a result of voluntary movements, and can thus gain knowledge about their sway in order to benefit from the information, these two possibilities cannot be distinguished in the empirical data, making it difficult to discriminate the cause-effect aspect of LT forces and balance.

Indeed, Riley [4] argued that reductions in postural sway that are measured in LT conditions could be an artifact of experimental conditions rather than true improvements in stability. Instructions in traditional LT conditions require participants to maintain their fingertip in a specific position and to apply a force that cannot exceed a small threshold, usually 1 N [1]. Riley refers to this as a supra-postural task: a requirement that guides behavior above and beyond that of maintaining upright balance. Participants may reduce postural sway in order to comply with this requirement. From this viewpoint the causal relation is reversed: postural sway is minimized in order to maintain LT, rather than as a result of information provided by LT. Consequently, in order to suitably investigate the effect of LT on balance, it is crucial to use an experimental paradigm that does not require an additional supra-postural task applied only in the LT conditions. The task given to the participants must be identical throughout the experiment.

To clarify the cause-effect relationship between LT and balance as well as to maintain the consistency of tasks in LT experiments, externally modulated LT may be utilized. Johannsen [3] studied balance in Parkinson’s Disease and stroke patients using LT that is externally applied and modulated by a therapist and not by the patient, which they called interpersonal touch (IPT). Unlike most other work in LT where the interaction force is experienced at the fingertip, IPT was applied at various locations on the backs of the patients by a therapist who was trained to apply only small interaction forces. The reduced postural sway reported in this work demonstrates the potential of externally applied LT as an effective and practical balance aid because it reduces the physical load on the therapist and may strengthen the patient’s ability to utilize his/her own sensory signals for balance rather than relying on external mechanical support.

More importantly, an experimental advantage is that IPT does not involve a supra-postural task for the perceiver, so any reductions in sway are true benefits. How then does the information contained in externally modulated LT compare to traditional paradigms? It is unclear from [3] how IPT was able to reduce postural sway because the interaction forces were not measured. One can speculate, however, that the applied LT was not strictly constant in magnitude, because constant LT cannot carry any information about sway. One can further speculate that the interaction force between two standing humans with non-zero sway (for both the patient and the therapist) cannot be strictly constant despite best efforts. The less-than-perfect modulation of force and/or the delay in the therapist’s reaction in maintaining the low level of force would result in variations in force magnitude that may be systematically related to the patient’s sway, and thus capable of providing useful information. In other words, the perhaps-unintentional modulation of light interaction force due to human motor control could have been the key to providing an additional sense of balance to the patients, thereby reducing postural sway.

Our objective in this study is to determine if a specific information about balance, encoded within the light interaction force, reduces postural sway. To study this, it requires the LT to be applied and modulated externally, similar to how the LT was applied externally in Johannsen et al. [3], but precisely modulated and monitored. For this, we used a haptic robot to provide measurable and precisely controlled LT on a participant’s back, imitating the role of the human therapist in Johannsen et al. [3].

The main hypothesis of this work is that LT reduces postural sway by providing additional information about one’s sway itself. That is, the IPT in Johannsen et al. [3] (or LT in general) is a form of biofeedback through small force modulation that provides postural sway information to humans. Similar to prior work in biofeedback [17, 18], we further hypothesize that the sway information provided by LT is either sway-velocity or sway-position dependent. To test this hypothesis, we have created one condition in which the modulated magnitude of LT is primarily velocity dependent, and a second condition in which a position dependent vibration is applied in addition to LT. This experimental design is further elaborated in the Methods section.

## Methods

### Experimental setup

**Fig 1:**
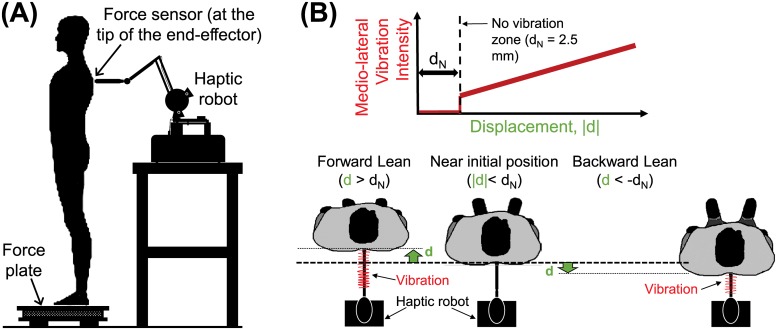
Experimental setup. (A) The haptic robot applies LT on the high back area of the participant, as the interaction force and the CoP is measured. (B) The position information from additional vibration feedback in the LTR+V condition. The no-vibration zone keeps excessive feedback onto the participant around the initial position, after which the intensity is proportional to the displacement.

Fig 1 depicts the experimental setup with a participant and the haptic robot. The participant is asked to stand barefoot in a bipedal stance on a force plate with their eyes closed while a robotic end effector is touching their high back on the spine near T3-T5 [3]. The Phantom robotic end effector (Phantom Premium 1.5/HF, 3D Systems) was equipped with a force sensor (Nano17, ATI Industrial Automation) where it touches the participant to monitor the varying force between the participant and the end effector. The force plate (Optima OPT400600HF, Advanced Material Technology Inc.) measured the ground reaction forces and moments at 1000 Hz, which were later used to calculate the CoP of the participant.

#### Sway-velocity information

**Fig 2:**
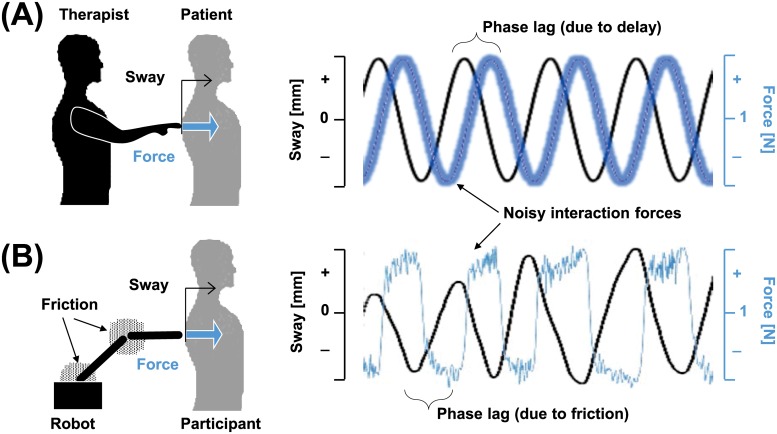
Externally modulated LT resembles the speculated force-to-sway profile. (A) The interaction force between the therapist and the patient in Johannsen et al. [3] is expected to be noisy as well as to lag the sway due to the neural processing delay. (B) The haptic robot is shown to provide similar force-to-sway profile due to friction in the robot joints.

We used this haptic robot to generate externally modulated light interaction forces that are intended to be similar to our best speculation of IPT in Johannsen et al. [3] (Fig 2). For humans, it would be challenging to maintain a constant force on a swaying patient due to several factors, including but not limited to feedback delay [19, 20]and inherent variability [21, 22] (Fig 2A). The delay would necessarily be greater than the reflex latency of 50~100 ms, and the actual cognitive modulation of precise force may result in a much greater delay [3]. It is speculated that the interaction force during IPT in Johannsen et al. [3] varies with the sway direction, with the applied force decreasing as the patient sways away from the therapist (forward) and vice-versa. Also, aside from the delay, human modulation of force will be naturally variable with noise.

Our haptic robot also does not supply a constant force to the participant due to friction and damping. The effect of damping forces that depend on the velocity appears similar to the effect of time delays in harmonic motion (Fig 2B). Also, the effect of variable static friction appears similar to the effect of natural variability in force control. Indeed, a robot that attempts to apply constant force under the forces from inherent damping and friction from robotic joints exhibit a similar profile to the speculated human-applied IPT with delay and variability (Fig 2). Hence, we expect a haptic robot with a constant modulation of force under damping/friction to exhibit an effect similar to IPT on human balance. This condition is referred to as Light Touch from the Robot (LTR). As a result of damping/friction, LTR condition is expected (and confirmed in the results section) to provide sway velocity-dependent information through small force modulation.

#### Sway-position information

To provide sway position-dependent information with externally applied LT, we note that by just by having our robot to lightly touch the participant, we inevitably provide the sway-velocity information to the participant due to static friction in the robot joints. That is, it is impossible to provide only sway position-dependent interaction forces without also providing sway velocity-dependent interaction forces. To mitigate this issue, we created another condition in which the haptic robot augments the LTR condition with a position-dependent vibration (LTR+V), similar to the vibrotactile feedback [17, 18]. In this condition, the haptic robot provides vibration in the mediolateral direction whose magnitude is proportional to the touch location’s distance from the center in the anterior-posterior direction (Fig 1B). Because the vibration is orthogonal to the light interaction force provided by the robot, we expect the position-dependency of the vibration to not interfere with the velocity-dependency of LT force provided by the LTR condition. However, in order to not provide excess vibration to minuscule sway, we implemented a 10-mm long no-vibration zone around the participant’s initial comfortable standing position (Fig 1B).

#### No additional information

To test the effect of velocity and position information on balance, the CoP data from the LTR and LTR+V conditions were compared to a baseline condition where no force was applied to the participant (NF). In NF condition, the robot did not touch the participant and no external additional information on body sway was provided to the participant.

### Procedure

Ten participants aged between 19 and 27 years (21.7 ± 2.3 years, 3 females, 7 males), participated voluntarily in the study. The individuals were healthy with no self-reported neuromuscular injuries or balance disorders. Prior to the experiment, all participants gave written consent as approved by the Missouri S&T Institutional Review Board (IRB). The participants were first made aware of the three experimental conditions, NF, LTR, and LTR+V. In all conditions, the participants were given identical instructions to stand as quietly as possible with their eyes closed. Instead of the light clothing in Johannsen et al. [3], which may affect the application of LT onto participants, our participants wore a skin-tight vest for the experiment to maintain the light interaction force on their body surface with high sensitivity and avoid disturbances due to loose clothes. In LTR condition, the participants were made aware of the force but were not instructed to maintain a specific force level nor to pay attention to the magnitude. In the LTR+V condition, the participants were informed that the vibration indicates the distance from the center in the anterior/posterior direction. The participants were given enough time to get comfortable with their stance on the force plate and instructed to sway as little as possible for the entire trial [1]. Each trial began when the participants felt stable enough and said ‘ready’ or ‘go’.

Each participant underwent 12 trials of each condition for a total of 36 trials per participant. These 36 trials were block-randomized into 3 blocks of 12 trials, where each block contained 4 trials of each condition. The first block was intended to make the participants accustomed to the LT by a robotic manipulator, whereas the second and third blocks were intended to capture the effect from conditions with sufficient number of trials. Each trial lasted 20 seconds [3], and mandatory breaks of five minutes were taken between blocks to minimize the effect from fatigue.

### Analysis

The ground reaction forces and moments of each participant were processed using MATLAB (MathWorks, 2018) to obtain the CoP data, which was smoothed with a moving average filter using a 100 ms window. Then, the anterior-posterior standard deviation of CoP (SD CoP) was obtained [23]. Also, the time history of CoP was differentiated to find the velocity of CoP (vCoP), from which the anterior-posterior standard deviation of vCoP (SD vCoP) was obtained [3]. The lateral component of CoP was not analyzed, as the light touch signal provided no information regarding velocity or position in this direction. For further discussion on the implications of these metrics, see the Discussion section.

To exclude possible learning effects, data from block 1 were not analyzed. In addition, to account for the high skew of the SD CoP and SD vCoP, statistical analyses were performed on the natural log transforms of these metrics instead (log(SD CoP) and log(SD vCoP)). The preliminary analyses leading to these decisions are presented in the results section. We used SPSS (IBM, version 23) to perform ANOVA with both participants and conditions as fixed factors. Tukey HSD was used to test the significance of the difference at *p* < 0.05.

In order to confirm the expected velocity-dependence of light interaction force from the robot in the LTR condition, a separate, single-trial experiment was conducted. While the haptic robot was set to the LTR condition, an experimenter gently pushed on to the end-effector while moving forward and backward multiple times to mimic the natural sway. The linear regression coefficient was found from the measured force-velocity relationship to verify the velocity-dependence.

## Results

The means and the standard deviations of SD CoP, as well as SD vCoP, are shown in Table 1. The values are comparable to other studies using these metrics [3, 23]. The average light interaction force applied by the haptic robot varied slightly by each trial due to individual differences but remained approximately 1 N±0.4 N, where the variation was due to friction and damping. For example, manual pushing against the robot with arbitrary motion resulted in interaction forces of 0.974±0.381 N.

**Table 1:** Measured balance metrics before log-transform.

	Condition	Mean±SD		Condition	Mean±SD
SD CoP [mm]	NF	4.81±2.26	SD vCoP [mm/s]	NF	12.83±7.14
	LTR	3.93±2.43		LTR	9.53±5.49
	LTR+V	3.17±1.27		LTR+V	10.39±5.48

We first looked for possible learning effects in our three conditions. While there were no significant differences between the blocks for the NF or LTR conditions, block 1 was significantly worse in the LTR+V condition for both SD CoP (p < 0.001) and SD vCoP (p < .0001), potentially indicating that it took participants longer to get acclimated to this condition. For this reason, we excluded all of the data from block 1, and ran all subsequent analyses using blocks 2 and 3.

Before the natural log transform, SD CoP showed skew of 1.66 and excess Kurtosis of 3.98, indicating highly asymmetric data distribution. Similarly, SD vCoP was highly asymmetric with skew of 2.26 and excess Kurtosis of 7.61. However, after log transform, both log(SD CoP) and log(SD vCoP) became much more symmetric, with both skew (0.10 and 0.72, respectively) and excess Kurtosis (-0.10 and 0.21, respectively) being within their acceptable ranges for statistical analysis using ANOVA. The resulting metrics of balance in this study are shown in Table 2.

**Table 2:** Log-transformed balance metrics with significance and effect sizes.

	Condition	Mean±SD	Significance	Cohen’s d
log(SD CoP)	NF	1.49±0.42		
	LTR	1.17±0.47	vs. NF: p < 0.001	vs. NF: d = 0.724
	LTR+V	1.02±0.45	vs. NF: p < 0.001,vs. LTR: p = 0.008	vs. NF: d = 1.085,vs. LTR: d = 0.323
log(SD vCoP)	NF	2.43±0.39		
	LTR	2.20±0.43	vs. NF: p < 0.001	vs. NF: d = 0.555
	LTR+V	2.24±0.43	vs. NF: p < 0.001,vs. LTR: p = 0.339	vs. NF: d = 0.453,vs. LTR: d = -0.100

**Fig 3:**
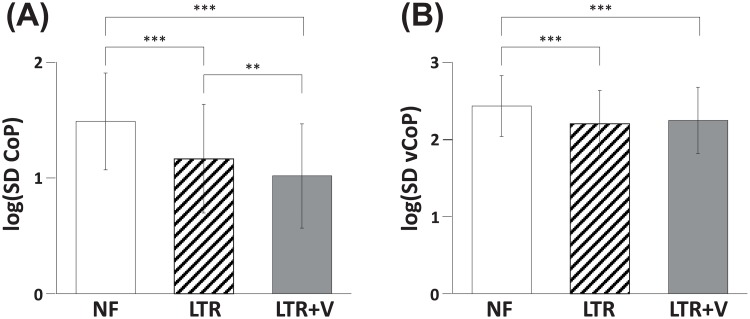
LT provided by the haptic robot improves standing balance. (A) The log(SD CoP) metric and (B) the log(SD vCoP) metric in NF (white), LTR (dashed) and LTR+V (gray) conditions. See Table 2 for details (***: p < 0.001, **: p < 0.01).

Sway variability was reduced due to the light interaction force applied by the haptic robot (Fig 3A). The log(SD CoP) was reduced from NF to LTR (p < 0.001) as well as from NF to LTR+V (p < 0.001). This metric was further reduced by the additional information about the trunk sway from LTR to LTR+V (p = 0.008). The log(SD vCoP) was reduced from NF to LTR (p < 0.001) as well as from NF to LTR+V (p < 0.001). However, no further improvement was found between LTR to LTR+V (p = 0.339). The trunk position information was shown to affect the log(SD CoP) metric only.

**Fig 4:**
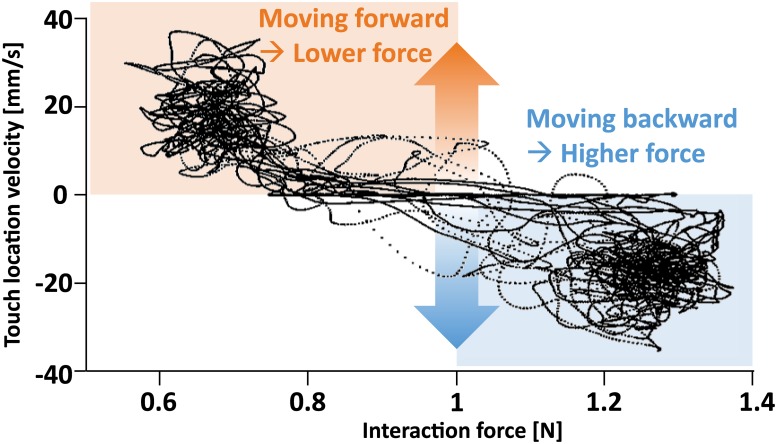
The interaction force between the haptic robot and the human versus the anterior-posterior velocity of the trunk at the touch location. The force-velocity correlation is R^2^ = 0.79.

Despite inconsistencies in force mapping, the interaction force applied by the haptic robot in the LTR condition was highly velocity-dependent (Fig 4, R^2^ = 0.79). The positive velocity of the contact point resulted in a lower magnitude of the contact force, whereas the negative velocity resulted in a larger contact force. The interaction force was also correlated to the position of the contact point but with comparably weaker dependency (R^2^ = 0.51).

## Discussion

This study is the first to show that externally modulated LT, similar to self-modulated LT, can reduce body sway. Such LT can be provided by a human [3] or by a robot as in this experiment, expanding the means to apply LT onto patients for balance treatment. In addition, this work supports the hypothesis that LT helps standing balance because it provides relevant information about one’s balance, where such information may include one’s trunk sway-velocity.

In this study, the participants had their eyes closed [1, 11, 14, 24, 25] to increase the difficulty of quiet standing in a healthy young population by blocking visual information [26, 27]. Tandem stance, another widely used means to increase the difficulty of standing in the mediolateral direction [1], was not used because our LT aimed at providing information regarding anterior-posterior body kinematics.

The decreased sway in the LTR condition may be due to the sway direction information inferable from the interaction force (Fig 2). When the participant sways forward (positive velocity), the end effector also moves forward to maintain the contact with the participant. In the process, the haptic robot has to work against static friction. As a result, the actual force applied to the participant is smaller than the commanded force until the sway velocity becomes zero. On the other hand, when the participant sways backward (negative velocity), the participant pushes against the commanded force as well as the static friction and damping. As a result, the actual force applied to the participant is larger than the commanded force until the sway velocity becomes zero. In essence, static friction enabled the LTR condition to provide a velocity-dependent interaction force to the participants.

Despite the limitations encountered in the experiment, such as the presence of static friction, externally modulated light interaction force provided by a haptic robot helped the participants to reduce their postural sway. It should be noted that static friction is not deterministically repeatable, as can be seen by the irregularities in Figs 2 and 4. As a result, the velocity dependence of the interaction force is also inconsistent, such that identical velocity does not produce identical interaction force. Nonetheless, the apparent velocity dependence of force was sufficient for the participants to significantly reduce their body sway. This suggests that precise modulation of the interaction force is not necessary for balance improvement. Indeed, even simple binary information about the sign of the velocity via the increase or decrease in the interaction force may be sufficient. This further suggests that the standing balance can be improved as long as the modulation of the interaction force (±0.4 N) is over the Weber fraction of the mean interaction force of (10~15% of 1 N) and encodes the direction of the sway velocity.

On the other hand, friction can tell the direction (not magnitude) of the movement, or the sign of the velocity, extremely fast and accurately. By definition, friction always resists movement. Because of this, the friction in the robot joints causes the pushing force to always decrease due to a movement away from the robot (forward sway velocity) and vice versa, providing accurate information about the direction of the movement at that exact time. In fact, if it is the only the sign (+ or -) of the velocity that we need, friction is more accurate and faster than any conceivable electromechanical realizations of a velocity sensor. Velocity derived either as the rate of change of position or the time integration of the acceleration suffers from noise and resolution in the measurement as well as the sampling time of digital circuitry, especially near zero velocity. In this regard, it is ironic that our “imperfect” haptic robot was in fact “perfect” in providing the direction of movement to the participants.

In the presented experiment, the instructed goal of our participants was to remain as quiet and stable as possible. That is, the participants were not instructed to pay attention to the interaction force. This was similar to the instruction given to the participants in [4] touching the curtain with his/her fingertip. In [4], this specific instruction resulted in no reduction of body sway, suggesting that the modification of balance from LT is a result of an additional supra-postural task implied by the experiment task–one which requires the participant to maintain a specific level of force against a specific position in space. However, unlike the supra-postural task in [4], participants in this study did not have to put additional effort to maintain contact, maintain the force level, nor to remain in a specific location in space. Nonetheless, the LTR and LTR+V conditions resulted in reduced log(SD CoP) and log(SD vCoP). This suggests that the decrease in body sway from LT cannot solely be from the additional supra-postural task described in [4] and that the brain successfully utilizes the information provided by LT for postural stabilization.

A distinguishing characteristic of our experimental design is that it allows for separating the LT provider (robot) and LT receiver (human participants). A notable benefit of using externally modulated light touch is that it makes it easier to characterize what information the participant is receiving and how. Unlike in traditional LT experiments where a participant both applies and receives LT [1, 2], Externally applied, modulated and measured LT can help investigate the cause-effect relationship of LT to balance.

A fingertip resting on a fixed surface could provide information about the current position of the trunk relative the finger, but it could also provide information about relative velocity or acceleration, and it is not clear which variable(s) are most useful for reducing sway. This observation leads to deeper questions regarding how the abstract concept of balance should be measured and quantified. One intriguing result is that the LTR+V condition was able to provide an improvement in log(SD CoP) but not log(SD vCoP), potentially suggesting separable cognitive modulation of balance that selectively affects these variables [18] (though this lack of difference could be a type II error). Theoretically, one can imagine balance strategies that selective optimize one variable but not the other. For example, a slow, drifting trajectory that meanders around the base of support could score well on the velocity metric while doing poorly on the position metric. Conversely, a series of rapid, small adjustments that confines the CoP to a small region near the center could show the opposite pattern. This potential divergence between two commonly used methods of quantifying stability indicates the need for a more nuanced, multidimensional approach.

While there are a number of different metrics for standing balance, those derived from the CoP are commonly used, especially in experiments that involve measuring the ground reaction forces. The physical model of upright human balance shows a direct relationship between the location of the center-of-mass (CoM) and the CoP of the support forces against the feet [28]. This relationship has made CoP-derived metrics popular for measuring postural sway, among which the standard deviations of the CoP and of CoP velocity are commonly used [29]. Each metric provides useful, though distinct information about postural sway and modulation [29, 30]. For example, greater CoP displacements and/or CoP velocities suggests a decreased ability to maintain postural control [29]. Indeed, CoP-derived metrics are reported to be able to detect increased fall risks [31], detect patients from healthy population [32], or to test factors affecting balance such as age, fear or fall history [33]. The CoP-derived metrics of balance are also critical parameters for developing accurate model for human balance modulation [29, 34–37]. In this regard, the CoP-derived metrics can be viewed as informative measures of balance.

Nonetheless, each of these measures has practical limitations to their usefulness [31, 38]. CoP displacement measures, such as the log(SD CoP) in this work, are prone to random overestimation due to shifting centers about which the body stabilizes, as any point within the support area outlined by the feet could reasonably be the point about which the body finds equilibrium at a given moment. CoP velocity measures, such as the log(SD vCoP) in this work, fail to be truly conclusive about balance control by themselves, as it is difficult to argue whether faster-but-smaller oscillations of the CoP are more (or less) stable than slower-but-larger oscillations. Furthermore, each metric likely measures distinct aspects of balance control [28, 31, 39, 40], thereby potentially showing significance in one metric but not another. When the two metrics are considered together, however, they account for the other’s weaknesses well and provide a more encompassing picture of balance control [4, 31]. Because of this, we report our findings in terms of both metrics. In this view, it is interesting to note that log(SD CoP) was sensitive to the trunk position information in the LTR+V condition but the log(SD vCoP) was not. It may be argued that the sway position metric, log(SD CoP), reflects the aspect of balance control that utilizes the sway position information, whereas the sway velocity metric, log(SD vCoP) does not (Fig 3A). One can also argue that the sway velocity metric, log(SD vCoP), fully reflects the aspect of balance that utilizes the sway velocity information only (Fig 3B). In other words, this work may have shown the existence of more than one aspect of balance modulation that can be measured only by the appropriate combination of metrics.

With regards to the position information provided in the LTR+V condition, it should be noted that although the light touch signal contains information about both the magnitude and direction of velocity, the vibrational component is identical for positions in front of and behind the center point, and thus only carries information about the magnitude and not direction. Future work could experiment with employing different directions of vibration (e.g. vertical vs. horizontal) depending on the sign of the displacement. It is speculated that these directions should be orthogonal to the direction of the velocity-dependent LT (fore-aft), because otherwise, the position-information would interfere with the velocity-information encoded in the LT. However, the efficacy of such differentiated vibration would depend on the participant’s ability to distinguish vertical versus mediolateral vibration at the touch location.
